# An unusual cause of headache: Expanding your differential

**DOI:** 10.1002/ccr3.9373

**Published:** 2024-09-03

**Authors:** Ijeoma Orabueze, Hazique Mohamed, Sehneet Grewal, Hector Ojeda‐Martinez

**Affiliations:** ^1^ Department of Internal Medicine Vassar Brothers Medical Center Poughkeepsie New York USA; ^2^ Department of Infectious Disease Vassar Brothers Medical Center Poughkeepsie New York USA

**Keywords:** *Coxiella*, giant cell arteritis, Q fever, temporal arteritis, vasculitis

## Abstract

Giant cell arteritis (GCA) is an inflammatory vasculitis that affects larger blood vessels, like the temporal arteries and the aorta. It is systemic and tends to affect individuals over 50. Common symptoms include headache, jaw pain provoked by chewing, and fever. We present an interesting case of a GCA variant.

## 1 INTRODUCTION

*Coxiella burnetii*, a less commonly known pathogen, has recently been recognized as a potential cause of giant cell arteritis (GCA). Although a less common etiological agent, it adds a layer of complexity in diagnosis and treatment, diverging from traditional GCA pathways predominantly linked to autoimmune mechanisms. It is suggested that the bacterium may trigger an abnormal immune response, leading to inflammation in the arterial walls.^1^ This case highlights the importance of considering a broad differential in atypical cases and the need for heightened clinical vigilance and comprehensive diagnostic approaches, particularly in patients presenting with non‐classical symptoms or an unusual epidemiological background. These insights are crucial for accurate diagnosis and effective treatment, broadening the scope of differential diagnoses in cases of arteritis.

## 2 CASE PRESENTATION

We present a 76‐year‐old healthy Israeli male who presented to the infectious disease clinic with complaints of generalized fatigue, headache, fever, and night sweats for about a two‐month duration. He described his headache as diffuse, superficial, non‐pulsating and endorsed tenderness over the temporal scalp. It was associated with fogginess, and he denied blurry vision or jaw claudication. Physical examination was negative.

## 3 INVESTIGATIONS AND TREATMENT

**FIGURE 1:**
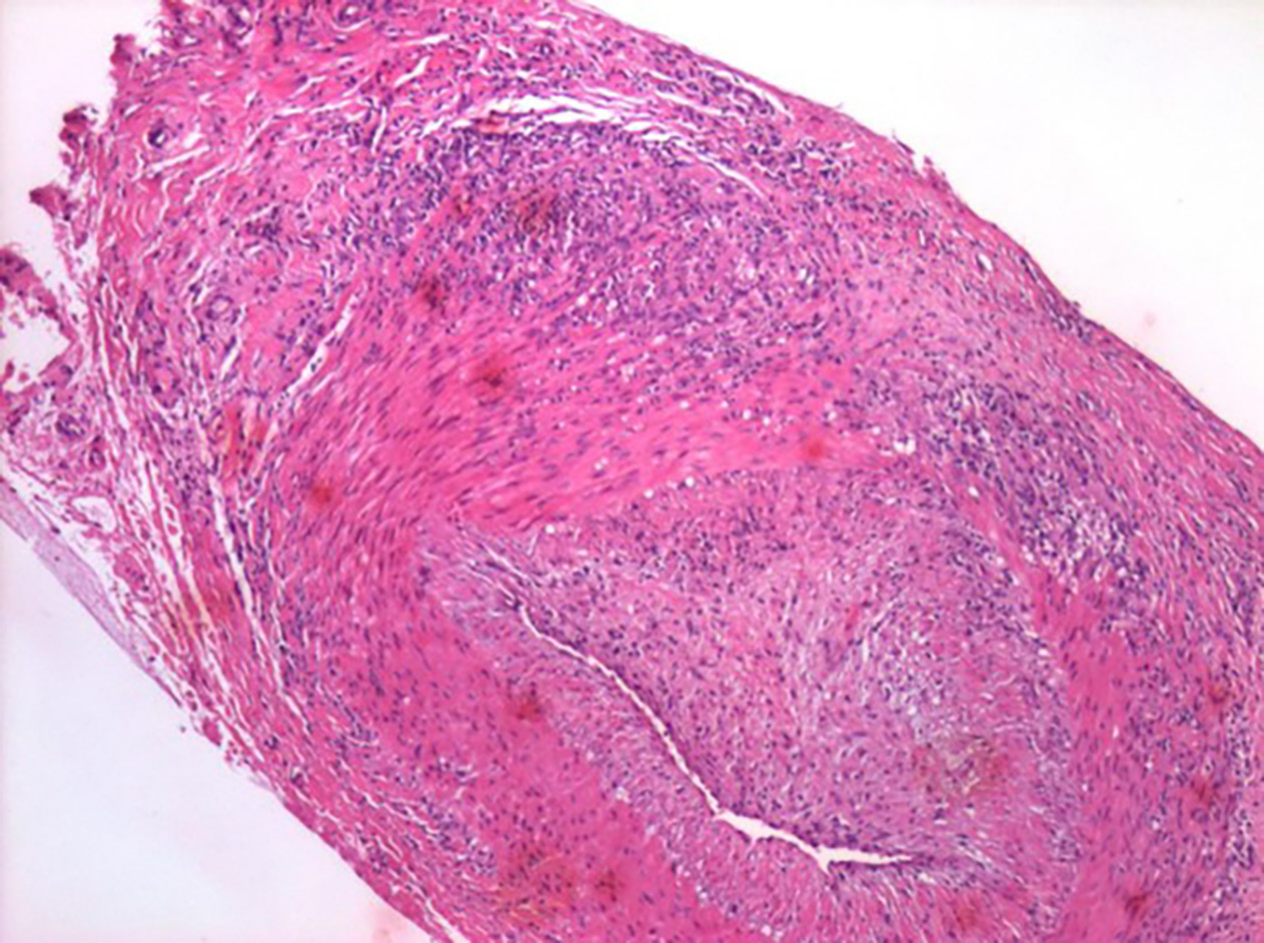
Left superficial temporal artery, biopsy: Vasculitis comprised of lymphohistiocytic and neutrophilic inflammatory cells. The infiltrate is focally transmural, extending from the adventitia to the internal elastic lamina. Areas of intimal inflammation are also identified. Definitive giant cells are not noted.

**FIGURE 2:**
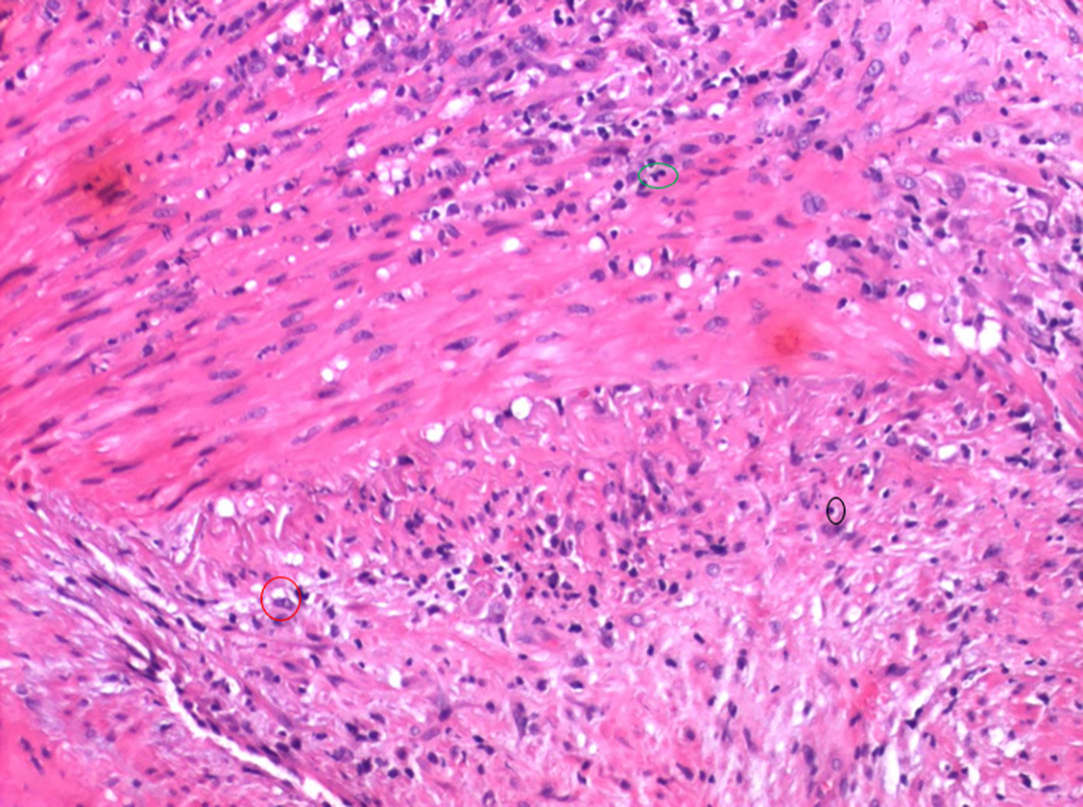
Higher power view of the inflammation showing a mixture of lymphocytes (black circle), histiocytes (red circle), and neutrophils (green circle).

**FIGURE 3:**
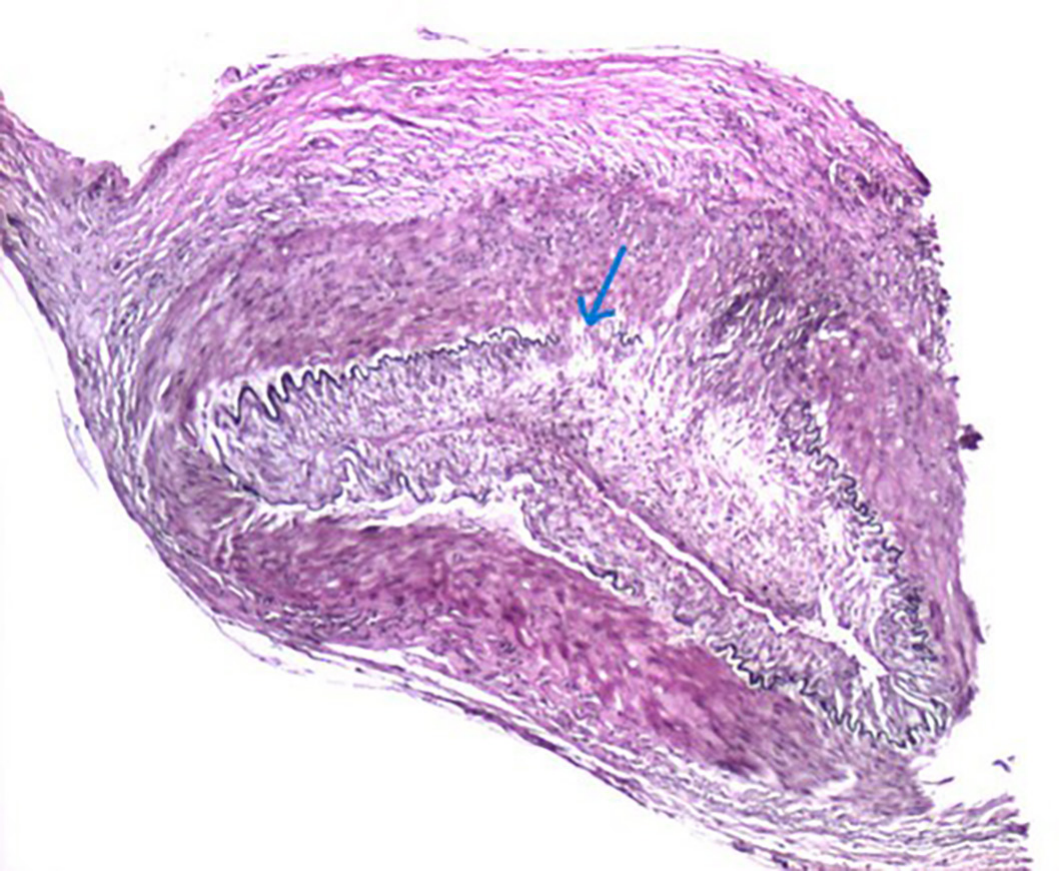
Elastic stain highlights the external elastic lamina and disruption thereof (arrow).

Laboratory values were pertinent for leukocytosis with neutrophilic predominance, normocytic anemia, thrombocytosis, ESR of 75 mm/h, and CRP 163 mg/L. He underwent a left temporal artery biopsy, which revealed vasculitis comprised of lymphohistiocytic and neutrophilic inflammatory cells (Figures 1, 2, 3). Extensive serological examinations were conducted to detect various pathogens, such as tuberculosis, Leishmania, Rickettsiae, Leptospira, HIV, West‐Nile Virus, Cytomegalovirus (CMV), and Epstein–Barr virus (EBV), to rule out any recent infections. Results indicated no evidence of acute infection except EBV and CMV, which were consistent with prior infection. MRI brain revealed a solitary non‐enhancing focus of a white lesion (0.6 × 0.5 cm) with a surrounding halo measuring 1.5 × 1.4 cm, non‐specific but pointing to subacute infarct, gliosis, or edema. Table 1 shows Phase I and Phase II antigen titers gotten a few weeks apart.

**TABLE 1:** Shows phase I and phase II antigen titers gotten a few weeks apart.

Day	Phase I antibody		Phase II antibody	
	IgM	IgG	IgM	IgG
1	ND	256	ND	2048
29	ND	256	ND	1024

Abbreviation: ND, not detected.

**Tissue sections of the temporal artery biopsy (**Figures 1, 2, 3 **)** exhibit eight complete cross‐sections of the muscular artery, two of which are extensively involved by prominent lymphohistiocytic inflammatory infiltrates with scattered neutrophils. The infiltrate is focally transmural, extending from the adventitia to the internal elastic membrane. Areas of intimal inflammation are also identified. Definitive giant cells are not noted. The described features are most consistent with temporal arteritis. While the absence of giant cells and the presence of neutrophils is unusual, the dominant pattern remains lymphohistiocytic vasculitis. The lack of fibrinoid necrosis and prominent small vessel inflammation argue against other vasculitides seen in the temporal artery, such as granulomatosis with polyangiitis and polyarteritis nodosa.

## 4 OUTCOME AND FOLLOW‐UP

At this point, he was referred to the rheumatology clinic. Upon further history taking, it was discovered that he had taken unpasteurized milk during his most recent trip to Israel. He was started on prednisone for suspicion of GCA. Serology testing for Coxiella was positive, indicating an acute infection and a convalescent serum was sent. Anticardiolipin IgM was elevated at 55MPL and doxycycline and Plaquenil were added to the regimen with immediate improvement in his symptoms. A PCR test was sent for Coxiella burnetti, and a CTA head and neck, ordered to rule out cerebral vasculitis was negative.

In the setting of symptom resolution and biopsy findings, weighing the risks and benefits, the decision was made to continue a tapered dose of steroids for the next 3 months, along with doxycycline 100 mg BID and hydroxychloroquine 200mg BID. Meanwhile, his temporal biopsy sample for PCR testing for Coxiella Brunetti returned negative. It has been over 4 months without symptom recurrence in the patient.

## 5 DISCUSSION

GCA, a large and medium‐sized vasculitis, has been associated with several infectious diseases.^2^ Our patient fulfilled three out of the four ACR criteria for GCA (age >50 years, tenderness of the temporal arteries, ESR >50 mm/h, and granuloma with multinucleated giant cells).^2^ While the exact cause of GCA is not fully understood, there have been suspicions that certain infectious diseases might trigger this harmful immune response.^3^ Q fever is caused by Coxiella burnetti, the intracellular organism that infects mononuclear phagocytes. It is most transmitted by inhalation of infected animal excreta and can rarely be transmitted through unpasteurized dairy products. According to the Center for Disease Control (CDC), the annual incidence of reported acute Q fever cases per million is one hundred and seventy‐eight.^4^ So far, we have encountered only five case reports on Coxiella‐induced temporal arteritis. In one of the cases, although the diagnosis was confirmed with a biopsy, the PCR analysis of the temporal artery biopsy was negative for *C. burnetii*, which was likely attributed to the initiation of antibiotic therapy before the biopsy.^5^

In acute Q fever, the phase I antibody should remain lower than phase II (Table 1). The phase II antibodies in our patient remained elevated (1:2048) in the initial sample and (1:1024) in the convalescent sample, causing high suspicion of acute infection. Acute Q fever commonly presents with debilitating headaches, nonspecific fever, chills, and myalgia. This clinical scenario prompts critical considerations regarding this patient's GCA pathogenesis and potential therapeutic strategies. A key point for deliberation is whether the presentation of GCA, in this case, was precipitated by *C. burnetii* infection or if it represents a transient expression of acute Q fever. Should the former be true, immediate inclusion of high‐dose corticosteroid therapy is imperative, as untreated GCA is associated with grave outcomes, including blindness, myocardial infarction, stroke, and dissecting aortic aneurysm, as delineated in the literature.^6^ Conversely, if the GCA is a transient manifestation of acute Q fever, resolving independently of corticosteroid intervention, it would prevent the need for long‐term steroid therapy and its associated complications, such as vertebral compression fractures, myopathy, and psychosis, among others. A combination of hydroxychloroquine and doxycycline is often used for acute Q fever patients at risk of chronic infection.^7^

Our patient's symptoms resolved within 2–3 days of management with prednisone, doxycycline, and Plaquenil. We maintained high clinical suspicion for Coxiella‐induced GCA despite the negative PCR due to the rapid improvement in symptoms upon treatment. Although PCR of the whole blood or serum can be positive soon after symptom onset, it usually becomes negative after antibiotic administration, as in our patient. In keeping with the recommendations from the CDC, treatment should be started based on the clinical picture and should not be withheld pending diagnostic tests.^4^

## 6 CONCLUSION

This case sheds light on GCA resulting from acute Q fever and its successful treatment with a combination of glucocorticoids and prolonged antibiotic therapy. The medical community recognizes high‐dose glucocorticoid therapy as the primary treatment for GCA, with intravenous methylprednisolone pulse therapy recommended for severe cases and conventional immunosuppressive drugs for refractory situations. Furthermore, Tocilizumab has been approved as an effective treatment for GCA. This case highlights the potential connection between Coxiella and the onset of GCA, provides valuable insights for the medical community, and instills hope for patients with GCA.

## AUTHOR CONTRIBUTIONS

**Ijeoma Orabueze:** Conceptualization; data curation; supervision; writing – original draft. **Hazique Mohamed:** Conceptualization; data curation; writing – original draft. **Sehneet Grewal:** Investigation; validation. **Hector Ojeda‐Martinez:** Formal analysis; investigation; supervision; visualization.

## FUNDING INFORMATION

None.

## CONFLICT OF INTEREST STATEMENT

The authors have no conflict of interest to declare.

## ETHICS STATEMENT

This case report was conducted in accordance with the declaration of Helsinki. The collection and evaluation of all protected patient health information were performed in a health insurance portability accountability Act (HIPAA)‐compliant manner.

## CONSENT

Written informed consent was obtained from the patient to publish this report in accordance with the journal's patient consent policy.

## Data Availability

The authors confirm that the data supporting the findings of this study are available within this article. Raw Data supporting the case's findings are available from the corresponding author upon reasonable request.
